# Supplementary material for: MiR-30a-5p Alters Epidermal Terminal Differentiation during Aging by Regulating BNIP3L/NIX-Dependent Mitophagy
Source: Cells. 2022 Feb 28;11(5):836. doi: 10.3390/cells11050836 (PMC8909909; doi:10.3390/cells11050836)
Supplement: Supplementary file 1 [file cells-11-00836-s001.zip › cells-1563567-supplementary.pdf]

Supplementary Materials & Methods

Supplementary Table S1. Specification of human biopsies used to generate primary keratinocytes.

Sample	Sex donor	Age donor	Anatomical location
Young 1	male	3	foreskin
Young 2	male	3	foreskin
Young 3	male	3	foreskin
Young 4	male	3	foreskin
Young 5	female	10	ear
Young 6	female	10	ear
Adult 1	female	26	breast
Adult 2	female	30	abdomen
Adult 3	male	36	abdomen
Adult 4	female	37	breast
Adult 5	female	44	abdomen
Adult 6	male	46	abdomen
Aged 1	female	68	breast
Aged 2	female	75	back
Aged 3	female	80	face
Aged 4	male	80	abdomen
Aged 5	female	92	buttock

Supplementary Table S2. List of primers pairs used for qPCR analysis and mutagenesis by reverse PCR.

Gene	Gene ID	Primer	Sequence
KRT10	3858	Forward	5′-TCCCCCTGATGTGAGTTGC-3′
		Reverse	5'-GAATCTGAATGACCGCCTGG-3′
IVL	3713	Forward	5'-GCAGTCATGTGCTTTTCCTCTTG-3′
		Reverse	5'-TCCTCCAGTCAATACCCATCAG-3′
LOR	16939	Forward	5'-TCATGATGCTACCCGAGGTTTG-3′
		Reverse	5'-CAGAACTAGATGCAGCCGGAGA-3′
TGM1	7051	Forward	5'-GAGAGCACCACACAGACGAG-3′
		Reverse	5'-GGGGTTGTTTCCGATGAGTA-3′
FLG	2312	Forward	5'-GCTGGAGTATTTTAGGAGATTCTGG-3′
		Reverse	5'-CTAGCCCTGATGTTGATATAGCCA-3′
KLK7	5650	Forward	5'-TTGGATCACATCAGATCCTCTCG-3′
		Reverse	5'-TAATCTTGTCACCCTGGGCTTC-3′
AQP9	366	Forward	5'-GTGAGGACCACAACAGGTAGG-3′
		Reverse	5'-GCCACATCCAAGGACAATCAAG-3′
CDSN	1041	Forward	5'-TCTCCTCCTGCCAGGGAC-3′
		Reverse	5'-CGTTAGGGGAGGTGATACGC-3′
BNIP3L	665	Forward	5'-TTGGATGCACAACATGAATCAGG-3′
		Reverse	5'-TCTTCTGACTGAGAGCTATGGTC-3′
TBP	6908	Forward	5'-TCAAACCCAGAATTGTTCTCCTTAT-3'
		Reverse	5'-CCTGAATCCCTTTAGAATAGGGTAGA-3'
RPL13A	6218	Forward	5'-CTCAAGGTCGTGCGTCTGAA-3'
		Reverse	5'-TGGCTGTCACTGCCTGGTACT-3'
HBB	3043	Forward	5'-CATCAAGCGTCCCATAGACTC-3′
		Reverse	5'-ACGTGGATGAAGTTGGTGGT-3′
SERPINA1	5265	Forward	5'-AAGGTGAGATCACCCTGACG-3′
		Reverse	5'-GTCAGTGAATCACGGGCATC-3′
MTND1	4535	Forward	5'-CAGAGACCAACCGAACCCC-3′
		Reverse	5'-GAAGAATAGGGCGAAGGGGC-3′
MTTL1	4567	Forward	5'-CACCCAAGAACAGGGTTTGT-3′
		Reverse	5'-TGGCCATGGGTATGTTGTTAA-3′

Mutagenesis		Primer	Sequence
BNIP3L	**Mut1**	Forward	5'-TGAATTAATGTACAGTCTTCCCAAGGTGATTCC-3′
		Reverse	5'-CTGTACATTAATTCAGTGAGAGATCAGAAGGC-3′
BNIP3L	**Mut2**	Forward	5'-TTTACACCAATTTGGGGACAAAAAGGCAGGC-3′
		Reverse	5'-CCAAATTGGTGTAAAGCTTTTTTAGC-3′
BNIP3L	**Mut3**	Forward	5'-TATATACAAATACATGTATAACTTGTAGCTATA-3′
		Reverse	5'-ATGTATTTGTATATAAAGCCTGCCTTTTTGT-3′

Supplementary Table S3. List of antibodies used for immunofluorescence and Western blot experiments.

	Target species	Host species	Dilution	Reference	Provider
**Immunofluorescence**	human BNIP3L	rabbit	1:200	HPA015652	Ozyme
	human KRT14	mouse	1:400	ab7800	Abcam
	rabbit IgG	goat	1:1000	A-11035	Thermo Fisher Scientific
	mouse IgG3	goat	1:1000	115-605-209	Jackson ImmunoResearch Ltd
**Western blot**	human BNIP3L	rabbit	1:400	HPA015652	Ozyme
	human VINCULIN	mouse	1:10 000	V9131	Sigma-Aldrich
	rabbit IgG	goat	1:10 000	1706515	Bio-rad
	mouse IgG	goat	1:10 000	1706516	Bio-rad

Supplementary Table S4. Specification of human biopsies used for immunostaining (selected tissue slides from the tissue microarrays SK244a and SK1001 from US Biomax). Donors in bold correspond to the tissue sections presented in Figure 3.

Tissue microarray ID	Sex donor	Age donor	Anatomical location
SK244a B3, B4	female	21	unknown
SK244a C1, C2	female	34	unknown
**SK244a C3, C4**	**female**	**38**	**unknown**
SKN1001 B3, B4	female	47	chest
SKN1001 D7, D8	female	40	chest
**SKN1001 D9, D10**	**male**	**35**	**chest**
SKN1001 E11, E12	female	40	chest
SKN1001 F11, F12	male	29	humeral back
SKN1001 G3, G4	male	19	groin
**SKN1001 G9, G10**	**female**	**42**	**anus**
SKN1001 H3, H4	male	33	anus
SKN1001 A11, A12	female	63	chest
SKN1001 B1, B2	female	62	chest
SKN1001 B11, B12	female	73	chest
SKN1001 C1, C2	female	73	chest
**SKN1001 C3, C4**	**female**	**71**	**chest**
SKN1001 C7, C8	female	71	chest
**SKN1001 E3, E4**	**female**	**61**	**chest**
SKN1001 F1, F2	female	64	chest
SKN1001 F7, F8	female	67	chest
**SKN1001 G1, G2**	**male**	**78**	**humeral back**

Real-time quantitative PCR program. All the primers have been designed to get an optimal annealing temperature at 60°C.

Step	Time	Temperature	Nb of cycles
Denaturation	30 sec	95°C	1
Denaturation	30 sec	95°C	40
Annealing	30 sec	60°C	
Elongation	30 sec	72°C	
Melting curve	1 min	95°C	1
	30 sec	60°C	
	+0.5°C / 30 sec from 60°C to 95°C		
